# Community pharmacists’ knowledge of, and role in, managing anticholinergic burden among patients with dementia in primary care: a cross-sectional survey study

**DOI:** 10.1007/s11096-024-01831-w

**Published:** 2024-11-25

**Authors:** Bara’a Shawaqfeh, Carmel M. Hughes, Bernadette McGuinness, Heather E. Barry

**Affiliations:** 1 Primary Care Research Group, School of Pharmacy, Queen’s University Belfast, 97 Lisburn Road, Belfast, BT9 7BL UK; 2 Faculty of Pharmacy, AL‐Zaytoonah University of Jordan, Amman, Jordan; 3 Centre for Public Health, Institute of Clinical Sciences, Royal Victoria Hospital, Queen’s University Belfast, Belfast, BT12 6BA UK

**Keywords:** Anticholinergic burden, Community pharmacies, Cross sectional survey, Dementia, Pharmacists

## Abstract

**Background:**

Anticholinergic medications and associated anticholinergic burden can impair cognitive function and increase mortality rates in patients with dementia.

**Aim:**

To explore community pharmacists’ knowledge of anticholinergic burden and perceptions of their role in anticholinergic burden management amongst patients with dementia.

**Method:**

A self-administered, postal questionnaire was distributed to all registered community pharmacies in Northern Ireland (n = 526) on two occasions (October and November 2022). The questionnaire comprised four sections: (1) demographics associated with pharmacists and pharmacies, (2) contact between community pharmacists and patients with dementia/their carers, and types of medication-related queries received by pharmacists, (3) understanding and knowledge about anticholinergic burden, and (4) community pharmacist role in management of anticholinergic burden in patients with dementia. Data were analysed descriptively using Stata v17.

**Results:**

A response rate of 15.2% (80/526) was achieved. Most contact was with patients with dementia/carers in their own homes. Community pharmacists lacked knowledge about anticholinergic burden in dementia, did not use any anticholinergic burden scales in their practice (n = 77, 96.3%), and rarely discussed anticholinergic burden with patients, carers (n = 60, 75.0%), or other healthcare professionals (n = 42, 52.5%). However, they were positive about their role in future interventions and saw value in managing anticholinergic burden in patients with dementia as part of a multidisciplinary primary healthcare team (n = 67; 83.3%).

**Conclusion:**

Despite the low response rate, the study findings have highlighted community pharmacists’ willingness to contribute to the management of anticholinergic burden in patients with dementia. Further research is required to understand how this can be achieved.

**Supplementary Information:**

The online version contains supplementary material available at 10.1007/s11096-024-01831-w.

## Impact statements

- Our findings suggest that community pharmacists in Northern Ireland would be willing to contribute to managing anticholinergic burden in patients with dementia as part of a multidisciplinary primary healthcare team.
- To support community pharmacists in extending their practice in this way, education and training would be needed to address current deficits.
- How community pharmacists could integrate with the multidisciplinary team needs to be considered.

## Introduction

Community pharmacists frequently encounter patients with dementia (PwD), especially those living in their own homes, and demonstrate positive attitudes towards them [1]. Community pharmacists deal with several issues in this patient population relating to over-the-counter medicine use, starting and stopping medications, and adherence-related queries [1]. A systematic review reported pharmacist-led medication management services for PwD may improve outcomes in both carers and patients [2]. However, there are gaps in healthcare professionals’ knowledge about dementia in general and anticholinergic medication use among PwD specifically. This is concerning given the association of anticholinergic burden (ACB; the cumulative effect of using multiple medications with anticholinergic properties concomitantly) with an increased risk of mortality in PwD as well as other negative outcomes such as cognitive and physical decline, neuropsychiatric disturbances, and institutionalisation [3, 4].

Some research has reported healthcare professionals’ knowledge about ACB; however, these studies (all conducted in the United Kingdom), predominantly focus on specialists in secondary care and rarely include pharmacists. Questionnaire studies with healthcare professionals [including geriatricians, general practitioners (GPs), nurses and gynaecologists] found knowledge about ACB in PwD was lacking [5, 6]. Another questionnaire study conducted in one area of England targeting primary and secondary care pharmacists reported that only a small proportion of respondents (15%) believed that dementia was a concern with anticholinergic medications [7]. Semi-structured interviews and focus groups conducted with healthcare professionals, patients and members of the public in Scotland indicated that participants believed pharmacists were well-placed to conduct reviews of anticholinergic medications, but overall responsibility for medication remained with the patient’s GP [8].

With community pharmacists being one of the most accessible healthcare professionals within primary care, they have a vital role in providing support about medicines-related issues to PwD and their carers, particularly in relation to ACB [9, 10]. However, in order to develop and deliver interventions that may target this, we need to understand community pharmacists’ knowledge of ACB, their current contact with PwD and their carers, and how they could contribute to the management of ACB in this patient population.

### Aim

This study aimed to explore community pharmacists’ knowledge of ACB and their perceptions of the role they could play in the management of ACB amongst PwD in Northern Ireland.

### Ethics approval

Ethical approval was granted by the Faculty of Medicine, Health and Life Sciences Research Ethics Committee, Queen’s University Belfast (27th October 2022, MHLS 22_143).

## Method

### Research design

A descriptive cross-sectional design was utilised. This study is reported according to the Consensus-Based Checklist for Reporting of Survey Studies.

### Questionnaire development

Questionnaire content was informed by the literature, previous work conducted by research team members, and research team discussions [1, 5, 7]. The questionnaire was piloted with eight pharmacists from the School of Pharmacy, Queen’s University Belfast to ensure face and content validity, and was estimated to take 10–15 min to complete.

The questionnaire comprised four sections (Supplementary File 1). Section A (seven items) collected non-identifiable demographic data about respondents and the community pharmacies in which they worked. Eight items in section B collected data about contact between community pharmacists, PwD and their carers and the medicines-related problems or queries community pharmacists commonly encountered whilst dealing with PwD. Respondents were asked to consider both PwD living in their own homes and those in care homes. Section C contained six items which explored community pharmacists’ understanding and knowledge of ACB in PwD. Finally, section D (six items) explored community pharmacists’ perspectives of the role they could play in the management of ACB in PwD.

### Participants and sample size

There are no publicly available contact details for individual community pharmacists in Northern Ireland. Therefore, questionnaires were distributed by post during October and November 2022 to all registered community pharmacies in Northern Ireland at the time of the study (n = 526) [11]. The questionnaire was to be completed by the pharmacist-in-charge/pharmacy manager to reduce response bias. This distribution method was utilised in previous studies conducted by the research team [1, 12], which achieved response rates greater than 30%.

Each questionnaire was distributed with an accompanying cover letter, summarising the study purpose, how to return the completed questionnaire and by which date (two weeks after the initial posting of the questionnaire). Approximately four weeks after the initial mailing, a reminder letter with a copy of the questionnaire was sent to all community pharmacies again as responses were completely anonymous and no respondent could be identified from the first mailing. Consent was implied if a pharmacist completed and returned a questionnaire [13].

### Data analysis

Responses from returned questionnaires were coded, entered into a customised Microsoft Excel spreadsheet [a random 10% sample double-checked by a second researcher (HB)], and imported into Stata v17 (Stata Corp LLC, College Station, Texas, USA) for analysis.

Descriptive analyses were used to describe the community pharmacist sample and responses. Previous studies had shown that demographic characteristics of pharmacists (e.g. holding a postgraduate qualification, number of years of experience) and the pharmacies in which they worked (e.g. having a contract to supply medication to a care home, pharmacy location) were associated with knowledge about dementia [1, 14, 15]. Therefore, it was planned to explore relationships between responses and these variables using Chi-squared analysis and Fisher’s exact test, with *p* ≤ 0.05 regarded as being statistically significant [1, 5].

For open-ended questions, responses were entered into Microsoft Word, analysed by one researcher (BS) using inductive content analysis [16, 17], and agreed through research team discussions. Respondent quotations supported interpretations, with a quotation identified by the letters CP (community pharmacist) and a number corresponding to the order of questionnaire receipt.

## Results

Sixty responses were returned after the first mailing, and twenty-one responses after the second. One returned questionnaire was blank, leaving 80 questionnaires with usable data for analysis [response rate = 15.2% (80/526)]. Given the low response rate, the planned inferential statistical analyses outlined could not be conducted.

### Demographic characteristics

Most respondents (n = 63, 79.0%) were aged between 25 and 54 years (Table 1). More than half (n = 42, 52.5%) had been qualified as a pharmacist for less than 15 years and working as a community pharmacist for less than 15 years (n = 42, 53.0%). Many respondents (n = 62, 77.5%) had no additional qualifications beyond their undergraduate pharmacy degree. Most respondents worked in a large chain (n = 32, 40.0%) or independent community pharmacy (n = 29, 36.3%) in urban or suburban areas.

**Table 1:** Demographic characteristics of respondents (n = 80)

Demographic data	N (%)
*Age (years)*	
> 25	6 (7.5)
25–34	25 (31.3)
35–44	22 (27.5)
45–54	16 (20.0)
55–64	9 (11.3)
< 65	0 (0)
Missing	2 (2.5)
*Number of years of qualification as a pharmacist*	
0 –5	20 (25.0)
6 –10	12 (15.0)
11–15	10 (12.5)
16 –20	10 (12.5)
21–25	8 (10.0)
> 25	18 (22.5)
Missing	2 (2.5)
*Number of years of working as a pharmacist in community pharmacy*	
0 –5	20 (25.0)
6 –10	12 (15.0)
11–15	10 (12.5)
16 –20	11 (13.8)
21–25	10 (12.5)
> 25	15 (18.8)
Missing	2 (2.5)
*Additional postgraduate qualifications*	
No	62 (77.5)
Independent prescribing	2 (2.5)
Certificate/ Diploma	5 (6.3)
MSc	3 (3.8)
PhD	2 (2.5)
Other	1 (1.3)
Missing	5 (6.3)
*Community pharmacy size*	
Independent	29 (36.3)
Small chain (group of < 5 pharmacies)	7 (8.8)
Medium chain (group of 5–20 pharmacies)	9 (11.3)
Large chain (group of > 20 pharmacies)	32 (40.0)
Missing	3 (3.8)
*Community pharmacy location*	
Rural (population < 5,000)	22 (27.5)
Suburban (population of 5,000 –10,000)	28 (35.0)
Urban (population > 10,000)	27 (33.8)
Missing	3 (3.8)
*Number of items (on average) dispensed on a typical weekday*	
< 100	2 (2.5)
100–199	11 (13.8)
200–400	41 (51.3)
> 400	23 (28.8)
Missing	3 (3.8)

### Community pharmacists’ contact with PwD and their carers

Nearly all respondents (n = 75, 93.8%) encountered PwD living in their own home. Of those, more than half, on average, (n = 39, 52.0%) dispensed medication for up to 14 PwD living in their own homes per month (Table 2). Pharmacists dealt with a family carer (n = 57, 76.0%) most often about the patient’s medicines. Common problems/queries related to adherence aids (n = 59, 78.7%), advice/counselling when starting new medications (n = 54, 72.0%), and patient non-adherence with medication (n = 52, 69.3%). Seven respondents (9.3%) provided additional details, broadly categorised as problems/queries relating to medication effects/side-effects, medication dosing and missed doses, ordering prescriptions from the GP and/or collecting medications from the pharmacy.

**Table 2:** Respondents’ contact with patients with dementia living in their own home (n = 75)

	N (%)
*On average, how many patients with dementia living in their own homes would you dispense medication for per month in the pharmacy in which you work?*	
< 5	5 (6.7)
5–9	15 (20.0)
10–14	19 (25.3)
15–19	12 (16.0)
20–24	4 (5.3)
≥ 25	18 (24.0)
Missing	2 (2.7)
*Concerning patients with dementia living in their own homes, whom would you tend to deal with most often about their medicines?*	
Patient themselves	7 (9.3)
Family carer	57 (76.0)
Professional carer (e.g. home help)	4 (5.3)
Nurse	0 (0)
GP	0 (0)
General practice pharmacist	1 (1.3)
Neighbour/Friend	0 (0)
Other	0 (0)
Spoiled response (respondent ticked more than one box)	6 (8.0)
*What problems/queries do you most commonly encounter whilst dealing with patients with dementia living in their own homes?*	
Advice/counselling when starting new medications	54 (72.0)
Queries about types of formulations available	29 (38.7)
Queries about patient non-adherence with medication	52 (69.3)
Queries about use of adherence aids	59 (78.7)
Advice about stopping medications	24 (32.0)
Request to complete review of patient medication	6 (8.0)
Advice on interactions with over-the-counter medicines	24 (32.0)
Information about non-pharmacological treatment options	6 (8.0)
No problems/queries	1 (1.3)
Other	7 (9.3)

Twenty-six respondents (32.5%) were working in a community pharmacy that had a contract to supply medication or advice to a care home. Of these, 17 (65.4%) were working in pharmacies that dispensed medication for up to 29 PwD per month, on average (Table 3). Respondents (n = 19, 73.1%) dealt with a nurse most often about care home residents’ medicines. Common problems/queries were about types of formulations available (n = 15, 57.7%), advice/counselling when starting new medications (n = 13, 50.0%), or stopping medications (n = 9, 34.6%).

**Table 3:** Respondents’ contact with care home residents with dementia (n = 26)

	N (%)
*On average, how many care home residents with dementia would you dispense medication for per month in the pharmacy in which you work?*	
< 9	8 (30.8)
10–19	5 (19.2)
20–29	4 (15.4)
30–39	1 (3.9)
40–49	2 (7.7)
≥ 50	6 (23.1)
*Concerning care home residents with dementia, whom would you tend to deal with most often about their medicines?*	
Resident themselves	0 (0)
Family member	0 (0)
Care assistant	6 (23.1)
Nurse	19 (73.1)
GP	0 (0)
General practice pharmacist	0 (0)
Neighbour/Friend	0 (0)
Other	0 (0)
Spoiled response (respondent ticked more than one box)	1 (3.9)
*What problems/queries do you most commonly encounter whilst dealing with care home residents with dementia?*	
Advice/counselling when starting new medications	13 (50.0)
Queries about types of formulations available	15 (57.7)
Queries about patient non-adherence with medication	2 (7.7)
Queries about use of adherence aids	2 (7.7)
Advice about stopping medications	9 (34.6)
Request to complete review of patient medication	4 (15.4)
Advice on interactions with over-the-counter medicines	2 (7.7)
Information about non-pharmacological treatment options	1 (3.9)
No problems/queries	4 (15.4)
Other	1 (3.9)

### Community pharmacists’ understanding and knowledge of ACB

**Fig. 1:**
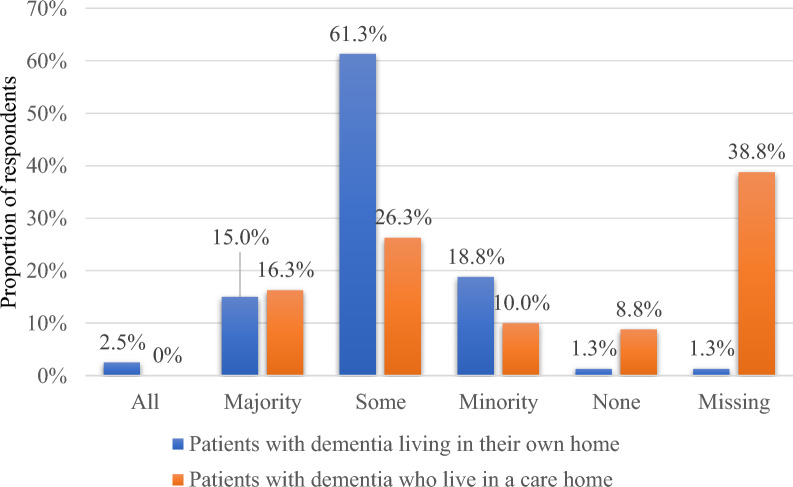
Proportion of patients with dementia living in their own homes and living in care homes that respondents considered to have high anticholinergic burden (ACB; n = 80 respondents)

Most respondents (n = 64, 80.0%) considered a ‘minority’ or ‘some’ PwD living in their own homes to have a high ACB whilst fewer respondents (n = 29, 36.3%) considered a ‘minority’ or ‘some’ PwD living in a care home to have a high ACB (Fig. 1).

Most respondents believed high ACB in PwD was related to an increased risk of falls (n = 73, 91.3%) and cognitive function decline (n = 64, 80.0%); fewer respondents believed ACB to be related to increased mortality rate (n = 24, 30.0%). Nearly all respondents (n = 77, 96.3%) reported that they did not use any scale to measure ACB. Three listed the Anticholinergic Burden Calculator [18], Anticholinergic Cognitive Burden Scale [19], and Anticholinergic Drug Scale [20] as scales that were used. Most respondents (n = 67, 84.0%) either strongly agreed or agreed that it was important for a community pharmacist to know the ACB of a PwD. The majority of respondents (n = 67, 83.8%) reported that knowing the ACB of a PwD would change how they managed that patient.

### Role of the community pharmacist in the management of ACB in PwD

Table 4 summarises respondents’ level of agreement/disagreement with statements presented in the final section of the questionnaire. Most respondents (n = 60, 75.0%) reported that they did not frequently provide advice to PwD about their ACB, yet nearly two-thirds (n = 49, 61.3%) believed discussing a patient’s ACB score with them or their carer/family member, was relevant to their role. More than half of respondents (n = 42, 52.5%) had not discussed a patient’s ACB with another healthcare professional. Many respondents (n = 56, 70.0%) agreed community pharmacists had an important role in management of ACB for PwD, with most (n = 67, 83.3%) agreeing they should be included in future interventions as part of a multidisciplinary team.

**Table 4:** Responses to statements regarding the role of the community pharmacist in the management of ACB among patients with dementia (n = 80)

	Strongly agree/ agree		Neither agree nor disagree		Strongly disagree/ disagree	
	N	%	N	%	N	%
I frequently provide advice to patients with dementia about their anticholinergic burden	7	8.8	13	16.3	60	75.0
I do believe that discussing a patient’s anticholinergic burden score with them, or their designated carer/family member is relevant to my role as a community pharmacist	49	61.3	21	26.3	10	12.5
I have discussed the anticholinergic burden of a patient with another healthcare professional	29	36.3	9	11.3	42	52.5
Community pharmacists have an important role in management of anticholinergic burden for patients with dementia	56	70.0	18	22.5	6	7.5
Community pharmacists should be included in future interventions to manage anticholinergic burden in patients with dementia as part of a multidisciplinary team	67	83.8	11	13.8	2	2.5

Twenty-four free-text comments were received about managing dementia patients’ ACB in community pharmacy. Several respondents noted that medication review was usually conducted in general practice, and their input was not required/needed:

*“Ultimately, prescribers dictate the clinical management of a patient and their conditions. Although our input is received graciously, the GP’s treatment rarely changes.”*(CP020)

In addition, many respondents reported a lack of resources to manage ACB for PwD in community pharmacies:

*“ECR [Electronic Care Record] is not available to our sector. We are trying to answer questions with all background [information] hidden from us.”(CP002) *

*“Greater support financially needs to be given if we are to do this [managing ACB in PwD] - not just more work for no pay.”*(CP027)

Respondents believed a key barrier was their own lack of knowledge about ACB and requested more training in this area:

*“I was unaware of this issue with regards to dementia patients prior to receiving this questionnaire.”*(CP039)

*“We need training [on how to manage ACB among PwD].”*(CP025)

However, respondents recognised that their accessibility to PwD and their carers was a strength, and they often had greater holistic knowledge about patients than other types of healthcare professional, which would be helpful in managing ACB in this patient population:

*“Community pharmacists are the only healthcare professionals where a patient can easily access information.”*(CP002)

*“Community pharmacists often know patients/families better than GPs. […] Can be involved in discussing risks by knowing more about how patients live, i.e. knowing how much help they [patients] have, who manages meds [medications], etc.”* (CP069)

## Discussion

This study found that community pharmacists lacked knowledge about ACB in dementia, did not use any ACB scales in their practice, and rarely discussed ACB with patients, carers, or other healthcare professionals. However, they were positive about their role and could see value in managing ACB in PwD as part of a multidisciplinary primary healthcare team.

The main strength of this study is its novelty since few studies have considered the community pharmacist role in this context. However, the low response rate limits the generalisability of the findings. This may have been due, in part, to strike action taken by postal workers in the national mail service during the questionnaire distribution period. Community pharmacists may have responded to the questionnaire only if they felt the topic was of interest or relevance to them, giving rise to non-response bias. Those who did respond were representative of younger pharmacists working in large chain community pharmacies in urban areas, with no postgraduate qualifications. Comparison between the respondent sample and wider pharmacist population was not possible despite efforts made by the researcher to obtain these data from the Pharmaceutical Society of Northern Ireland (the regulatory and professional body for pharmacists in Northern Ireland; C Connolly, 2023, personal communication). The small sample size meant that descriptive analyses only were undertaken; it is not known if respondent characteristics (e.g. holding additional postgraduate qualifications or their experience with PwD) may have affected their response.

Almost all respondents reported having contact with PwD living in their own home, predominantly through a family carer, which reflects other research conducted with community pharmacists [1, 21]. This finding is not unexpected as the majority of PwD live at home and wish to remain living there for as long as possible [22, 23]. Carers are acknowledged to play a crucial role in supporting PwD, particularly as patients’ cognitive and physical functioning decline [24, 25] and have been described as the ‘invisible second patient’ [25, 26]. Previous research has shown family carers of PwD experience significant burden, emotional distress and reduction in quality of life [27, 28], with management of medicines highlighted as being a main source of stress [29]. Respondents reported that queries about the use of adherence aids were frequently encountered when dealing with PwD living in their own homes. Medication adherence can be problematic for PwD [31–34]. A study conducted by members of the research team reported that both GPs and community pharmacists believed medication adherence was poor amongst PwD, and community pharmacists believed they had a role in monitoring adherence in PwD particularly in over-adherence to medication [35]. A recent systematic review found a range of effective interventions for self-management of medicines by PwD living in their own home and their family carers, e.g. reminder devices [36]. Carers’ medication management skills, their knowledge of the disease, and understanding of dementia medications could also improve patients’ adherence to treatment [37]. Community pharmacists may be considered ‘guardians of adherence’ for patients with chronic conditions [38–41] and have an important role in promoting adherence in PwD and supporting carers with medicines management.

Fewer respondents were working in a community pharmacy that had a contract to supply medication or advice to a care home. Those that did most often dealt with a nurse regarding care home residents’ medicines, indicating little direct community pharmacist contact with care home residents with dementia which is supported by earlier research [1]. In the current study, community pharmacists dealt with queries about medication formulation types available for care home residents with dementia, particularly with cases of dysphagia (difficulty with swallowing) [42]. Alternative formulations such as oro-dispersible tablets, patches, or liquid formulations can be offered, depending on availability [42–44].

Community pharmacists considered the majority of PwD to not have high ACB. This contradicts findings from epidemiological studies which reported a high prevalence of ACB, and anticholinergic medication use among PwD [45]. Anticholinergic medication use in people with cognitive impairment has nearly doubled over a 20-year period [46] and presence of dementia was associated with an increasing ACB score [47]. In this study, it was reassuring that community pharmacists were able to identify many risks associated with high ACB in PwD. However, it was surprising that few respondents associated increased mortality rate with high ACB; a recent observational study using Northern Irish data, highlighted that higher ACB was associated with significantly higher mortality rates in PwD in comparison to PwD who had no ACB [3]. This would indicate that community pharmacists may not be aware of the prevalence of ACB amongst PwD or the most recent evidence in this area, especially regarding the risks of ACB. Undergraduate education for pharmacists must include core topics relating to medicines optimisation for older people and highlight the specific challenges when prescribing and providing pharmaceutical care for PwD including the risks associated with the use of anticholinergic medications. Further postgraduate educational provision would allow pharmacists to gain enhanced training depending on their area of clinical practice or clinical expertise.

Only three community pharmacists reported using ACB scales but could see their value and believed that management of ACB in PwD was part of their role. Using these scales in community pharmacy could potentially identify PwD at high risk from both prescription and non-prescription (over-the-counter) medications which may contribute to ACB and identify those who would benefit from medication review. Consequently, this may improve outcomes such as cognitive functioning and quality of life [30, 48–52]. Community pharmacists reported they did not discuss ACB with PwD, their carers, or other healthcare professionals. This may result from their lack of knowledge about ACB, lack of access to patients’ medical records, time constraints and staffing issues within the pharmacy, which has been reported in other research on medicines management in PwD [35]. Furthermore, Araklitis and colleagues reported pharmacists lacked confidence in discussing ACB [7]. Campbell and colleagues demonstrated that pharmacists could successfully reduce exposure to high-risk anticholinergic medications in older adults in primary care, although PwD were not included in this work [53]. Further work is needed to develop multidisciplinary interventions promoting accessibility of community pharmacists to identify PwD with high ACB and needing medication review.

Due to the low response rate, further research is needed to corroborate the study findings, possibly through in-depth qualitative work with community pharmacists. Respondents highlighted significant gaps in their knowledge about ACB in PwD which could be addressed through the development of an educational intervention. Future work should also focus on closing communication gaps between primary healthcare professionals who routinely measure ACB (e.g. GPs) and others who may be able to utilise this information in their clinical practice, such as community pharmacists.

## Conclusion

Despite the low response rate, unique insights into community pharmacists’ knowledge of ACB and perceptions of their contribution in the management of ACB amongst PwD in Northern Ireland were obtained. Primary care is a key setting to deliver effective interventions to reduce ACB among older people and PwD in particular. There is therefore an opportunity for community pharmacists, as experts in medicines and accessible healthcare professionals within primary care, to focus on and lead this issue, making a positive contribution to caring for this unique patient population.

## Supplementary Information

Below is the link to the electronic supplementary material.

Supplementary file1 (DOCX 82 KB)
