# Supplementary material for: Community pharmacists’ knowledge of, and role in, managing anticholinergic burden among patients with dementia in primary care: a cross-sectional survey study
Source: Int J Clin Pharm. 2024 Nov 25;47(2):345–53. doi: 10.1007/s11096-024-01831-w (PMC11919982; doi:10.1007/s11096-024-01831-w)
Supplement: Supplementary file 1 — Supplementary file1 (DOCX 82 KB) [file 11096_2024_1831_MOESM1_ESM.docx]

**
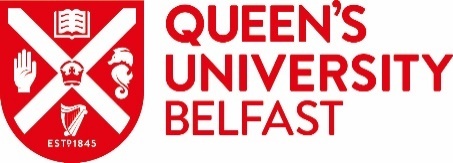
**

**A cross-sectional questionnaire study of community pharmacists’ knowledge of and role in managing anticholinergic burden among patients with dementia in primary care in Northern Ireland**

| **Section A** | Demographic information |
| --- | --- |
| **Section B** | Contact with patients with dementia and their carers |
| **Section C** | Understanding and knowledge of anticholinergic burden |
| **Section D** | Role of the community pharmacist in the management of anticholinergic burden in patients with dementia |

| **How to complete this questionnaire**  The aim of this questionnaire is to explore the knowledge of community pharmacists about anticholinergic burden among older patients (i.e. those aged 65 years and older) with dementia in primary care and their perceptions of their role in managing anticholinergic burden in this patient population in Northern Ireland.  In this study, we are focusing on patients with dementia living in their own homes or in a care home (i.e. nursing or residential home), so please bear this in mind as you complete the questionnaire.  This questionnaire should take approximately 10-15 minutes to complete. There are no right or wrong answers, and all answers are useful. Participation in this study is completely voluntary. By completing this questionnaire, you are consenting to participate in this study.  The information collected in this study will be used for research purposes only and may be presented at conferences or in academic journals. Responses will be treated in strict confidence. As the questionnaire is completed anonymously, it will not be possible to link you with the responses provided. |
| --- |

| **Section A: Demographic information** | | | | | | | | | | | | | | | |  |
| --- | --- | --- | --- | --- | --- | --- | --- | --- | --- | --- | --- | --- | --- | --- | --- | --- |
| *This section of the questionnaire seeks to collect details about you and the community pharmacy in which you work. Please answer by placing a tick in the appropriate box.* | | | | | | | | | | | | | | | | |
| **Which category below includes your age?** | | | | | | | | | | | | | | | | |
| <25 | 25 - 34 | | | | | 35 - 44 | | | 45 - 54 | 55 – 64 | | | | ≥65 | | |
| 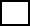 | 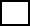 | | | | | 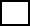 | | | 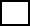 | 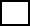 | | | | 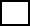 | | |
| **How many years have you been qualified as a pharmacist?** | | | | | | | | | | | | | | | | |
| 0 - 5 | | 6 - 10 | | | 11 - 15 | | | 16 - 20 | | | 21 – 25 | | | | >25 | |
| 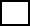 | | 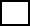 | | | 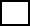 | | | 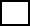 | | | 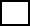 | | | | 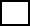 | |
| **How many years have you been working as a pharmacist in community pharmacy?** | | | | | | | | | | | | | | | | |
| 0 - 5 | | 6 - 10 | | | 11 - 15 | | | 16 - 20 | | | 21 – 25 | | | | >25 | |
| 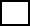 | | 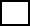 | | | 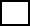 | | | 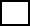 | | | 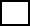 | | | | 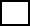 | |
| **Do you hold any additional postgraduate qualifications?** | | | | | | | | | | | | | | | | |
| No | Independent prescribing | | | | | Certificate/ Diploma | | | MSc | PhD | | | | Other | | |
| 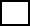 | 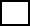 | | | | | 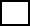 | | | 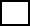 | 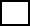 | | | | 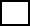 | | |
| **If you answered ‘other’, please provide further details about these additional qualifications below:** | | | | | | | | | | | | | | | | |
|  | | | | | | | | | | | | | | | | |
| **Which of the following best describes the community pharmacy in which you work?** | | | | | | | | | | | | | | | | |
| Independent | | | Small chain  (group of <5 pharmacies) | | | | Medium chain  (group of 5-20 pharmacies) | | | | | | Large chain  (group of >20 pharmacies) | | | |
| 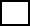 | | | 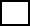 | | | | 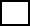 | | | | | | 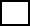 | | | |
| **How would you best describe the location of the community pharmacy in which you work?** | | | | | | | | | | | | | | | | |
| Rural  (population <5,000) | | | | Suburban  (population of 5,000 – 10,000) | | | | | | | | Urban  (population >10,000) | | | | |
| 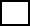 | | | | 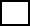 | | | | | | | | 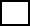 | | | | |

| **How many items (on average) would the pharmacy in which you work dispense on a typical weekday?** | | | |
| --- | --- | --- | --- |
| <100 | 100 - 199 | 200 - 400 | >400 |
| 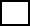 | 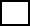 | 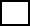 | 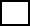 |

| **Section B: Contact with patients with dementia and their carers** | |
| --- | --- |
| *This section of the questionnaire seeks to examine the contact you have with patients with dementia and their carers.* | |
| **Do you encounter patients with dementia living in their own homes?** | |
| Yes | No |
| 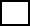 | 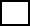 |
| **If you answered NO to question 8, please go straight to question 12.**  **If you answered YES to question 8, please continue below.** | |
| **On average, how many patients with dementia living in their own homes would you dispense medication for per month in the pharmacy in which you work?** | |

| <5 | 5 - 9 | 10 - 14 | 15 - 19 | 20 - 24 | ≥25 |
| --- | --- | --- | --- | --- | --- |
| 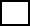 | 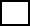 | 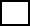 | 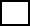 | 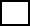 | 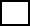 |

| **Concerning patients with dementia living in their own homes, whom would you tend to deal with most often about their medicines?**  **(Please tick only ONE option)** | | |
| --- | --- | --- |
| 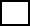 | Patient themselves | |
| 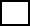 | Family carer | |
| 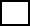 | Professional carer (e.g. home help) | |
| 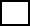 | Nurse | |
| 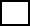 | GP | |
| 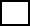 | General Practice Pharmacist | |
| 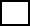 | Neighbour/Friend | |
| 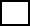 | Other (please state): | |
|  | | |
| **What problems/queries do you most commonly encounter whilst dealing with patients with dementia living in their own homes?**  **(Please tick ALL that apply)** | | |
| 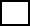 | Advice/counselling when starting new medications | |
| 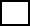 | Queries about types of formulations available | |
| 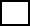 | Queries about patient non-adherence with medication | |
| 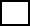 | Queries about use of adherence aids | |
| 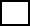 | Advice about stopping medications | |
| 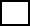 | Request to complete review of patient medication | |
| 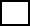 | Advice on interactions with over-the-counter (OTC) medicines | |
| 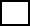 | Information about non-pharmacological treatment options | |
| 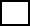 | No problems/queries | |
| 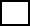 | Other (please state): | |
|  | | |
| **Do you have a contract with a care home to supply medication or advice?** | | |
| Yes | | No |
| 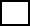 | | 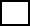 |
| **If you answered NO to question 12, please go straight to question 16.**  **If you answered YES to question 12, please continue below.** | | |
| **On average, how many care home residents with dementia would you dispense medication for per month in the pharmacy in which you work?** | | |

| ≤9 | 10 - 19 | 20 - 29 | 30 - 39 | 40 – 49 | ≥50 |
| --- | --- | --- | --- | --- | --- |
| 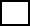 | 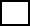 | 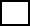 | 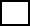 | 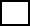 | 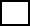 |
| **Concerning care home residents with dementia, whom would you tend to deal with most often about their medicines? (Please tick only ONE option)** | | | | | |

| 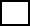 | Resident themselves |
| --- | --- |
| 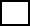 | Family member |
| 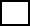 | Care assistant |
| 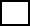 | Nurse |
| 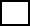 | GP |
| 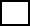 | General Practice Pharmacist |
| 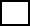 | Neighbour/Friend |
| 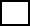 | Other (please state): |
|  | |

| **What problems/queries do you most commonly encounter whilst dealing with care home residents with dementia? (Please tick ALL that apply)** |
| --- |

| 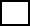 | Advice/counselling when starting new medications |  |
| --- | --- | --- |
| 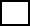 | Queries about types of formulations available |  |
| 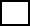 | Queries about patient non-adherence with medication |  |
| 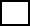 | Queries about use of adherence aids |  |
| 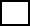 | Advice about stopping medications |  |
| 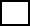 | Request to complete review of patient medication |  |
| 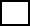 | Advice on interactions with over-the-counter (OTC) medicines |  |
| 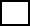 | Information about non-pharmacological treatment options |  |
| 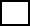 | No problems/queries |  |
| 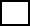 | Other (please state): | |

|  | | | |
| --- | --- | --- | --- |
| **Section C: Understanding and knowledge of anticholinergic burden** |  |  |  |
| *This section of the questionnaire seeks to explore your understanding and knowledge of anticholinergic burden in patients with dementia. Please think about older patients with dementia (those aged ≥65 years) living in their own home or in a care* *home, when answering these questions.* | | | |
| The term **‘anticholinergic burden’** refers to the cumulative effect of using multiple medications with anticholinergic properties at the same time.  Anticholinergic burden can be measured, and several scales have been developed to produce a ‘score’ for anticholinergic burden such as the Anticholinergic Risk Scale (ARS), Anticholinergic Drug Scale (ADS), Anticholinergic Cognitive Burden (ACB), Drug Burden Index (DBI), and Anticholinergic Loading Scale (ALS). These scales rate medications according to their anticholinergic activity from 1 (low anticholinergic activity, such as warfarin and levocetirizine) to 3 (high anticholinergic activity, such as olanzapine and oxybutynin). Many studies have reported that patients with dementia have a high anticholinergic burden, and that this is linked to a range of negative effects. | | | |
| **What proportion of your patients with dementia living in their own homes would you consider as having high anticholinergic burden? (Please tick only ONE option)** | | | |

| All | Majority | Some | Minority | None |
| --- | --- | --- | --- | --- |
| 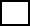 | 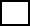 | 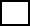 | 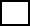 | 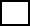 |

| **What proportion of your patients with dementia who live in a care home would you consider as having high anticholinergic burden? (Please tick only ONE option)** |
| --- |

| All | Majority | Some | Minority | None |
| --- | --- | --- | --- | --- |
| 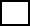 | 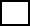 | 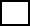 | 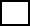 | 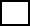 |

| **Considering the list below, please select the item(s) that you believe is/are related to high anticholinergic burden in patients with dementia. (Please tick ALL that apply)** | |
| --- | --- |
| 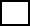 | Decline in physical function |
| 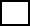 | Increased risk of falls |
|  | Reduced quality of life |
|  | Decline in cognitive function |
|  | Increased mortality rate |
|  | Hospitalisation |

| **Do you use any scale to measure anticholinergic burden in your pharmacy practice? (Please tick only ONE option)** | |
| --- | --- |
| Yes | No |
|  |  |
| **If you answered Yes, please provide the name of this scale(s) below:** | |
|  | |

| **Please indicate your level of agreement with the following statement by placing a tick in the appropriate box:**  **It is important for a community pharmacist to know the anticholinergic burden of a patient with dementia.** |
| --- |

| Strongly agree | Agree | Neither agree nor disagree | | Disagree | Strongly disagree |
| --- | --- | --- | --- | --- | --- |
|  |  |  | |  |  |
| **Do you feel that knowing the anticholinergic burden of a patient with dementia would change how you manage that patient?**  **(Please tick only ONE option)** | | | | | |
| Yes | | | No | | |
|  | | |  | | |
| **Please indicate why you chose this answer:** | | | | | |
|  | | | | | |

| **Section D: Role of the community pharmacist in the management of anticholinergic burden in patients with dementia** | | | | | |
| --- | --- | --- | --- | --- | --- |
| *This section of the questionnaire seeks to explore your perspectives about the role community pharmacists can play in managing anticholinergic burden for patients with dementia. Please indicate your level of agreement with the following statements by placing a tick in the appropriate box.* | | | | | |
|  | Strongly  agree | Agree | Neither agree nor disagree | Disagree | Strongly disagree |
| **I frequently provide advice to patients with dementia about their anticholinergic burden.** |  |  |  |  |  |
| **I do believe that discussing a patient’s anticholinergic burden score with them, or their designated carer/family member is relevant to my role as a community pharmacist.** |  |  |  |  |  |
| **I have discussed the anticholinergic burden of a patient with another healthcare professional.** |  |  |  |  |  |
| **Community pharmacists have an important role in management of anticholinergic burden for patients with dementia.** |  |  |  |  |  |
| **Community pharmacists should be included in future interventions to manage anticholinergic burden in patients with dementia as part of a multidisciplinary team.** |  |  |  |  |  |

| **Do you have any further comments about managing dementia patients’ anticholinergic burden in community pharmacy?** |
| --- |
|  |

**Thank you for taking the time to complete this questionnaire; your participation is much appreciated. Please place your completed survey in the pre-paid addressed envelope and return it to the research team.**
